# Reverse transcription progression and genome length regulate HIV-1 core elasticity and disassembly

**DOI:** 10.1371/journal.ppat.1013269

**Published:** 2025-06-12

**Authors:** Akshay Deshpande, Jiong Shi, Noa Rotem-Dai, Christopher Aiken, Itay Rousso

**Affiliations:** 1 Ben-Gurion University of the Negev, Department of Physiology and Cell Biology, Beer-Sheva, Israel; 2 Vanderbilt University Medical Center, Department of Pathology, Microbiology and Immunology, Nashville, Tennessee, United States of America; University of Wisconsin, UNITED STATES OF AMERICA

## Abstract

The structural and mechanical properties of the HIV-1 core are critical for successful infection, balancing stability for early replication and controlled disassembly for genome release. Recent studies have highlighted the role of core elasticity in nuclear entry, yet the molecular determinants regulating this property remain poorly understood. Here, atomic force microscopy (AFM) was used to investigate the relationship between reverse transcription progression, genome length, core elasticity, and disassembly. The results demonstrate that reverse transcription induces a gradual loss of elasticity, rendering the core increasingly brittle as DNA synthesis progresses. Cores containing shorter genomes remained highly elastic, whereas those with longer genomes exhibited increased brittleness, structural damage, and a higher degree of disassembly, after 4 hours of reverse transcription. Additionally, cores from an RNase H-deficient HIV-1 mutant retained high elasticity. These findings provide insight into the interplay between genome synthesis, core integrity, and nuclear entry, supporting a model in which reverse transcription-generated mechanical stress facilitates uncoating. Furthermore, early-stage reverse transcription preserved core elasticity, suggesting a temporal window for successful nuclear import before structural destabilization compromises infectivity.

## Author summary

Successful HIV-1 infection requires that the viral core remain intact during cytoplasmic trafficking and undergo controlled disassembly within the nucleus to enable genome release. In this study, atomic force microscopy was used to investigate how the progression of reverse transcription and viral genome length influence the mechanical properties of isolated HIV-1 cores. The results show that core elasticity is maintained during early stages of reverse transcription but progressively declines as DNA synthesis advances, leading to increased brittleness and capsid rupture. Additionally, cores containing shorter genomes remained structurally intact and mechanically resilient following reverse transcription, whereas cores encoding longer or wild-type genomes exhibited extensive disassembly and mechanical failure. Collectively, the data support a model in which mechanical stress generated during reverse transcription gradually compromises core integrity, defining a critical spatiotemporal window in which the core must retain sufficient elasticity to traverse the cytoplasm and enter the nucleus before disassembly impairs infectivity.

## Introduction

The HIV-1 core is a dynamic structure comprising a conical shell formed by hexameric and pentameric arrangements of capsid (CA) proteins. This shell encapsulates the viral genomic RNA, as well as the reverse transcriptase (RT) and integrase (IN) enzymes, which are critical for the replication cycle [1,2]. The structural and mechanical properties of the HIV-1 core are finely tuned, as they must provide sufficient stability to protect the viral genome and enzymes during early stages of infection, while also enabling regulated disassembly during later stages [3–5].

The stability of the HIV-1 core is essential for its infectivity, with mutations in the CA protein that either stabilize or destabilize the core significantly impairing viral replication [4,6,7]. This highlights the critical balance between stability and flexibility required for successful infection. Recent evidence underscores the importance of maintaining core integrity for efficient reverse transcription, a process wherein the viral RNA genome is converted into double-stranded DNA (dsDNA) within the core [8–11]. However, for infection to proceed, the core must undergo a controlled disassembly process known as uncoating, ultimately releasing the reverse-transcribed viral dsDNA for integration into the host genome [12–14].

Uncoating is a tightly regulated process influenced by both viral and host cellular factors. Host proteins such as inositol hexakisphosphate (IP6), cyclophilin A (CypA), cleavage and polyadenylation specificity factor 6 (CPSF6), transportin 3 (TNPO3), and tripartite motif-containing protein 5α (TRIM5α) have been shown to interact with the capsid and modulate core stability [2,15–18]. These interactions not only influence the timing of uncoating but also contribute to the efficiency of reverse transcription and subsequent integration. Importantly, studies suggest that successful completion of reverse transcription is closely tied to the disassembly of the viral core, although the precise trigger for uncoating remains poorly understood.

Experimental studies from our group have demonstrated that reverse transcription mechanically induces uncoating in vitro [19,20]. Specifically, distinct stages of reverse transcription were correlated with specific mechanical disruptions of the core structure. Based on these findings, we proposed a mechanistic model for HIV-1 core uncoating, wherein reverse transcription acts as a mechanical driver of disassembly. In this model, the progression of reverse transcription induces mechanical perturbations in the capsid lattice, resulting in structural fractures that accumulate over time. This accumulated damage ultimately leads to complete disassembly of the core [19]. More recently, independent studies have corroborated these findings, lending further support to the hypothesis that accumulated mechanical damage plays a central role in HIV-1 uncoating [9,21,22].

Until recently, HIV-1 uncoating was thought to occur in the cytoplasm of the target cell [23,24]. However, more recent evidence suggests the persistence of an intact core that traverses the nuclear pore complex (NPC) and undergoes uncoating inside the nucleus in close proximity to transcriptionally active sites in the host DNA [14,25–31]. This shift in understanding highlights the importance of an intact capsid for nuclear entry and subsequent integration. The requirement of an intact capsid for the core to pass through the NPC is particularly challenging, given the comparable size of the core and the nuclear pore [25,32–35].

In a recent study [36], we demonstrated that native HIV-1 cores are highly elastic, and that property plays a key role in nuclear entry. HIV-1 cores capable of entering the nucleus and infecting the cell were shown to withstand extreme deformation without undergoing structural damage. In contrast, several mutant viruses exhibiting impaired nuclear entry were observed to break following forced compression. These results suggest that the extreme elasticity of the core allows it to traverse through the tight nuclear pore while remaining intact, enabling successful infection. This finding provides critical insight into the unique mechanical properties of the HIV-1 core and their role in viral replication.

In this study, we show that HIV-1 core elasticity depends on its structural integrity. As the capsid becomes more damaged during reverse transcription, the core is less capable of withstanding forced compression without undergoing breakage. Furthermore, the extent of core damage and uncoating are directly linked to the progression of reverse transcription and the length of the reverse-transcribed dsDNA within the intact core. Our results suggest a requirement for spaciotemporal control of reverse transcription to retain capsid elasticity required for nuclear entry, followed by completion of reverse transcription and capsid breakage within the nucleus.

## Results

### Reverse transcription-induces changes inWT HIV-1 cores elasticity and brittleness

Previous studies employing atomic force microscopy (AFM) revealed the mechanism of reverse transcription-induced uncoating of HIV-1 cores [19,20]. These studies identified three distinct stiffness spikes occurring during the reverse transcription process. The first spike occurred within the initial 10–30 minutes of reverse transcription, followed by two subsequent spikes appearing at 40–80 minutes and 120–160 minutes. These stiffness transitions culminated in the complete disassembly of the core after 5 hours of reverse transcription.

Building upon this observation, the current study employed a previously developed AFM technique to investigate the impact of reverse transcription on the elasticity and brittleness of isolated HIV-1 cores [36]. Cores were prepared as described in earlier protocols [19,20,36–38], and reverse transcription was initiated by adding MgCl_2_ (1 mM) and dNTPs (100 μM). Reverse transcription was halted at predefined time points (30, 60, 120, 180, and 240 minutes) by addition of the inhibitor Efavirenz (100 nM). These time points were selected to correspond to the previously identified stiffness spikes, with the 240-minute time point representing the longest interval before the near-complete disassembly of cores, which precluded elasticity measurements.

To evaluate core elasticity during reverse transcription, the percentage of cores that fractured under compressive force was quantified at each time point. Compression was applied to a defined region within the main body of the core by scanning it with a high loading force of 5 nN, which induced substantial local deformation. The force was then rapidly reduced to 300 pN, and the compressed region was imaged repeatedly over time. Volume changes within the scanned region were quantified as a function of time. The volume beneath the scanned region was calculated over time to assess elastic response. At the end of each the experiment (7–10 minutes), the entire core was imaged to identify structural damages resulting from compression.

**Fig 1:**
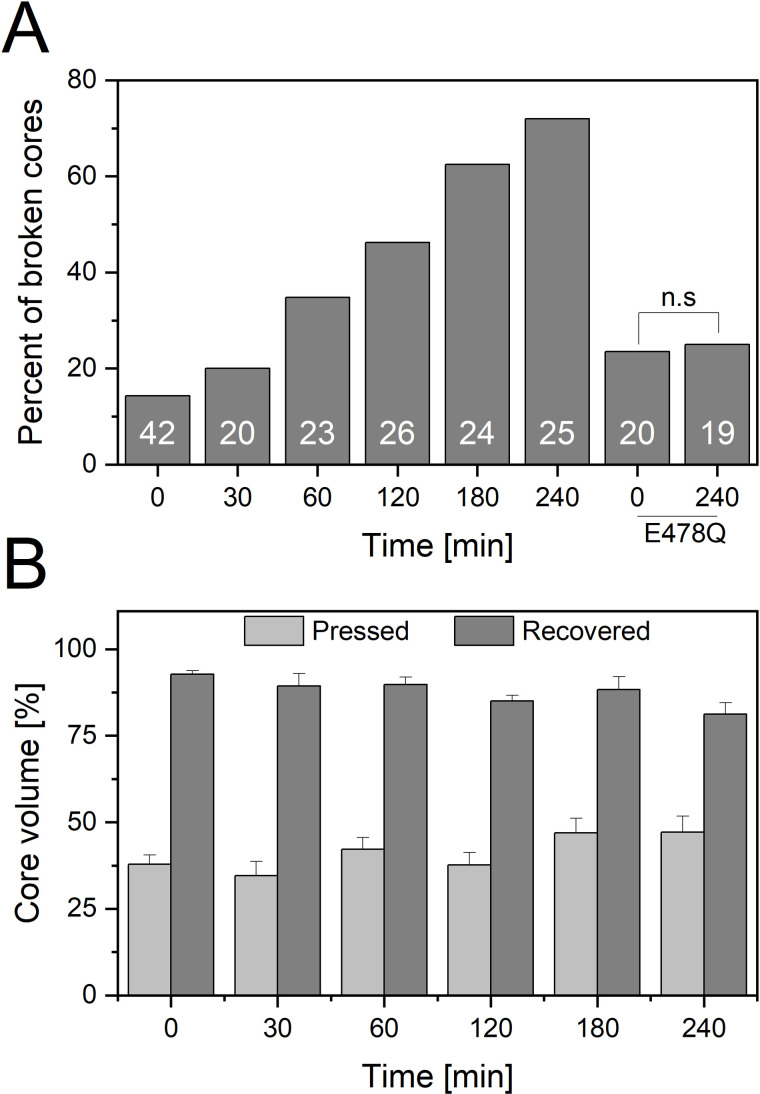
Reverse transcription progression increases HIV-1 core brittleness. **(A)** Percentage of HIV-1 cores that fractured under compressive force at different time points during reverse transcription. Cores containing WT genome, were examined in the presence of IP6 (100 μM). Reverse transcription was initiated by adding MgCl_2_ (1 mM) and dNTPs (100 μM) and halted at specific time points using Efavirenz (100 nM). Brittleness of WT cores exhibited a strong correlation with reverse transcription duration (R² = 0.983). A Chi-square test for independence showed that the difference in brittleness of E478Q cores between 0 and 240 minutes was not statistically significant. The number of cores analyzed is indicated inside each bar. Core breakage was determined by AFM imaging of the cores after they were compressed by a loading force of 5 nN. **(B)** The average volume recovery of the cores analyzed in **A.** The compressed volume (Pressed) was obtained by scanning a region on the cores surface at a relatively high force (5 nN). The volume upon reducing the force back to 300 pN is labeled as Released. T-test analysis revealed that the difference between the compressed and the recovered volume is significant (p value <0.0001). Error bars represent the standard error of the mean.

Fig 1 illustrates the relationship between reverse transcription time and two parameters: the proportion of broken cores containing WT genome (Fig 1A) and the average percentage of volume recovery following compression (Fig 1B). Broken HIV-1 cores were defined as capsids exhibiting structural abnormalities, including distortion of their native-like morphology, visible damage to the lattice surface of the main body, or damage at one or both ends of the core. In contrast, intact cores retained their characteristic morphology without detectable defects. Prior to the initiation of reverse transcription, 14% of cores fractured under compression, indicative of high elasticity. During the early stages of reverse transcription, the cores remained predominantly elastic. However, as reverse transcription advanced, a gradual increase in core brittleness was observed, culminating in 72% of cores fracturing at the 240-minute time point. In parallel, a slight reduction in average volume recovery was noted over the course of reverse transcription. Specifically, 93 ± 1% of the original volume was recovered prior to reverse transcription, compared to 81 ± 3% after 240 minutes. Despite this modest decline, volume recovery remained high throughout the entire time course, suggesting that local elastic properties of the capsid shell were largely retained, even as global structural integrity deteriorated.

To further dissect the relationship between reverse transcription progression and core elasticity, we analyzed HIV-1 cores from an RT mutant defective in RNase H activity (E478Q). This mutant supports early steps of reverse transcription, including the synthesis of minus strand strong stop DNA, but is impaired in minus strand transfer [39]. Notably, cores from the E478Q mutant retained high elasticity, with minimal damage observed even after 240 minutes of reverse transcription. These results are consistent with the elasticity observed in WT cores during the early stages of reverse transcription, thus supporting the finding that early reverse transcription maintains core elasticity.

**Fig 2:**
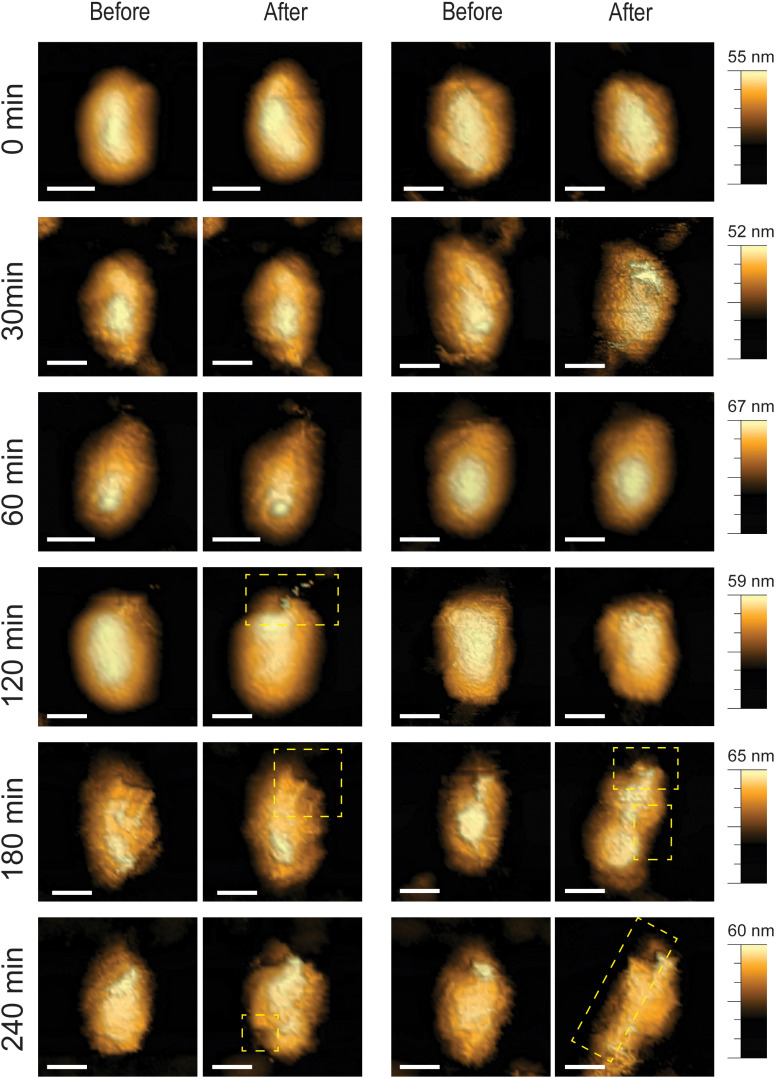
Reverse transcription induces progressive structural damage to HIV-1 cores. Topographic AFM images of isolated IP6-treated cores before and following high-force (5 nN) compression. At early time points, damage was localized to one end of the core, whereas later time points exhibited more extensive structural disruption. All images were acquired using the QI mode at a maximal loading force of 300 pN. For clarity, openings or damages in the cores are shown within a dashed yellow rectangle. Scale bars are 50 nm.

**Fig 3:**
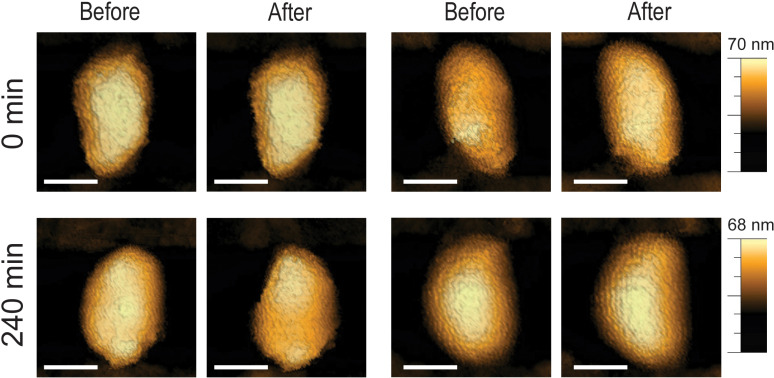
RNase H-deficient HIV-1 mutant cores retain high elasticity despite prolonged reverse transcription. Representative AFM images of E478Q cores treated with IP6 at 0 min and 240 minutes of reverse transcription, showing minimal structural damage in E478Q cores. Images were acquired using the QI mode at a maximal loading force of 300 pN. Scale bars are 50 nm.

Moreover, morphological analysis revealed that reverse transcription progression not only increased the percentage of cores fracturing under compression but also amplified the extent of structural damage. At early time points, core breakage was confined to one end of the structure (Fig 2). By contrast, at later stages of reverse transcription, the damage became more extensive, affecting the entire core. However, analysis of the RNase H-deficient E478Q mutant cores (Fig 3) showed that, even after 240 minutes of reverse transcription, these cores remained structurally intact following compression. This observation reinforces the conclusion that early-stage reverse transcription preserves core elasticity and structural integrity, whereas continued genome synthesis progressively weakens the capsid, ultimately promoting genome release.

### Reverse transcribed genome length determines core uncoating and elasticity

Previous studies conducted in our laboratory demonstrated that reverse transcription mechanically induces uncoating and that termination of reverse transcription at early stages preserves core integrity, as observed in the RNase H-deficient E478Q mutant [19]. The current investigation aimed to determine the length of reverse-transcribed dsDNA required for uncoating and its effect on core elasticity.

**Fig 4:**
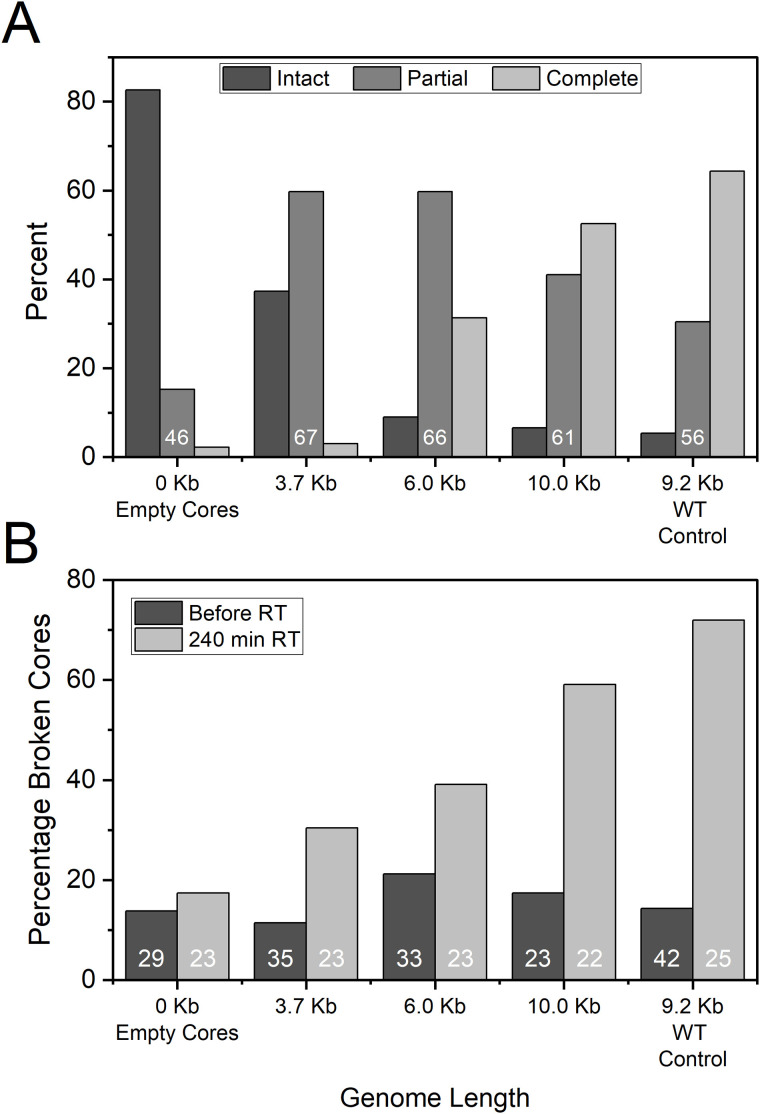
Genome length influences HIV-1 core uncoating and elasticity. **(A)** Assessment of core disassembly after 6-7 hours of reverse transcription in virions packaging genomes of different lengths (3.7 kb, 6.0 kb, 10.0 kb, and WT 9.2 kb). Cores were classified as intact, partially disassembled, or fully disassembled. The total number of cores analyzed in each genome length is labeled. **(B)** Elasticity measurements of HIV-1 cores before and after 240 minutes of reverse transcription. Elasticity was assessed by measuring the percentage of cores that fractured under compressive force. A weak correlation between core brittleness and genome size was observed prior to reverse transcription (R^2^ = 0.023), which strengthened significantly after 240 minutes of reverse transcription (R^2^ = 0.884). Data for WT-control was taken from Fig 1. The number of cores analyzed in each group is displayed within the corresponding bar.

**Fig 5:**
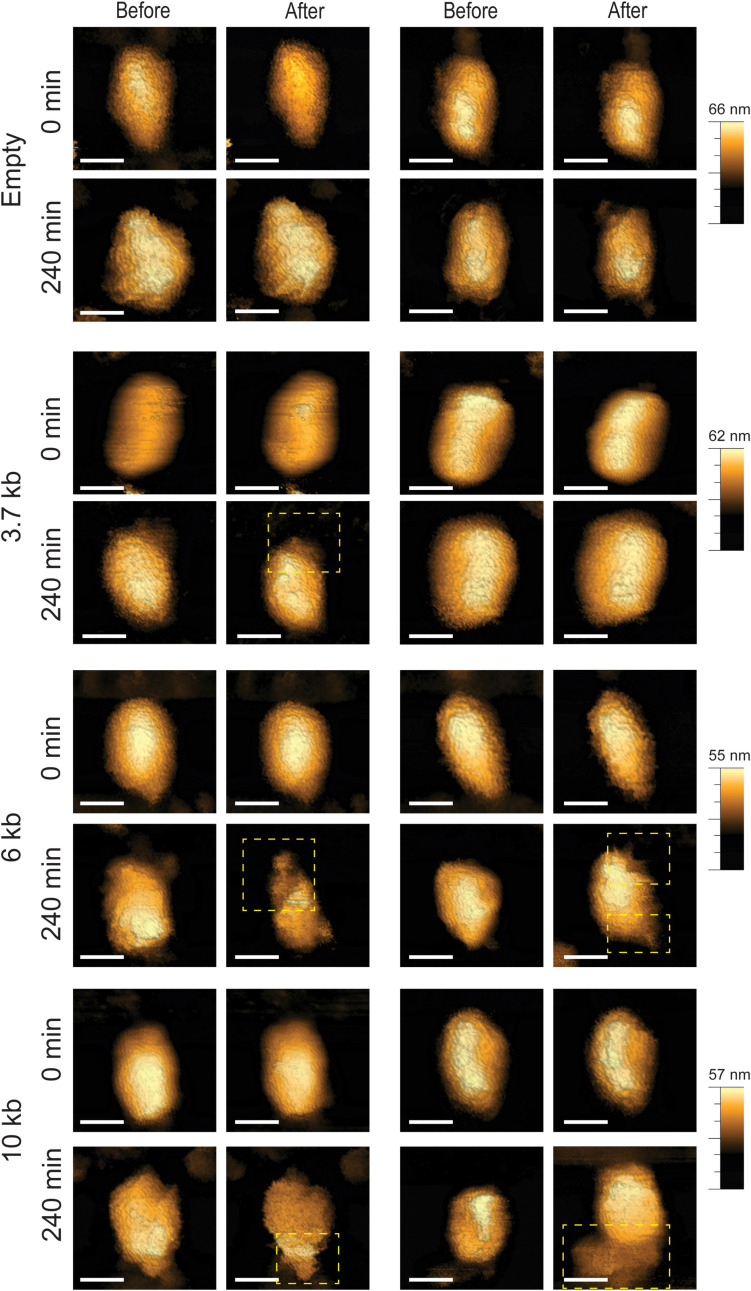
Representative topographic AFM images of IP6-treated cores before and after compression as a function of genome length. Images were acquired at 0 min and 240 min of reverse transcription using QI mode with a maximal loading force of 300 pN. Prior to reverse transcription, cores remained intact following compression. After 240 min of reverse transcription, cores packaging longer genomes exhibited increased disassembly, reducing their ability to withstand compression and resulting in greater structural damage. For clarity, openings in the cores are shown within a dashed yellow rectangle. Scale bars, 50 nm.

To address this, WT cores containing RNA genomes of varying lengths were prepared using a lentiviral packaging system. Specifically, cores were produced with no genome (empty cores), or with shortened genomes of 3.7 kb and 6.0 kb. In addition, cores containing a 10.0 kb genome—which is comparable in length to the native viral genome—were included. As a control, WT cores containing the native 9.2 kb genome were used. The structural integrity of cores was analyzed after 6–7 hours of reverse transcription (Figs 4 and 5). Cores were categorized into three groups based on morphology: intact (complete and native-like structure), partially disassembled (loss of no more than 25–30% of structure), and nearly fully disassembled cores (shown broken fragments or residual debris of cores).

As shown in Fig 4A, most genomeless cores remained intact (83%) after 6 hours of reverse transcription, with a minor fraction (15%) exhibiting partial damage. Reverse transcription of cores containing a 3.7 kb genome drastically reduced the proportion of intact cores to 37%, with the majority (60%) showing only partial damage. Increasing the genome length to 6.0 kb resulted in a similar proportion of partially damaged cores (60%), but the percentage of intact cores decreased further to 9%, accompanied by an increase in fully disassembled cores (30%).

Cores containing a 10.0 kb genome exhibited a small fraction of intact cores (5%) after reverse transcription, with the majority transitioning to either partial disassembly (41%) or complete disassembly (52%). Similarly, WT cores with a 9.2 kb genome showed a comparable percentage of intact cores (7%). Notably, despite the slightly shorter genome length in WT cores, the efficiency of complete disassembly was higher, with 64% of cores undergoing full disassembly and 30% showing only partial disassembly.

In a second set of experiments, the elasticity of HIV-1 cores was evaluated before and after reverse transcription to investigate the impact of genome length on core mechanics (Fig 4B). Measurements conducted prior to reverse transcription revealed that core elasticity was unaffected by genome length, showing no significant differences among cores containing genomes of varying lengths. Empty cores, as well as those packaging genomes of 3.7 kb, 6.0 kb, 10.0 kb, and wild-type (WT) HIV-1 cores (9.2 kb genome), all exhibited high elasticity like WT cores. These results indicate that genome length does not affect the inherent elasticity of intact cores prior to reverse transcription.

The elasticity of the cores was subsequently measured after four hours of reverse transcription, which was terminated by the addition of efavirenz. Empty cores retained their high elasticity, consistent with the absence of reverse transcription. Similarly, cores containing a 3.7 kb genome exhibited only a slight reduction in elasticity, while the proportion of broken cores increased modestly from 11% to 29%.

In contrast, cores containing longer genomes exhibited progressively greater reductions in elasticity. Cores packaging genomes of 6.0 kb and 10.0 kb demonstrated significant losses in elasticity, with broken core percentage increasing to 39% and 59%, respectively. Interestingly, despite containing a slightly shorter genome length than 10 kb, WT cores exhibited the most pronounced loss in elasticity, with the proportion of broken cores peaking at 72% after 240 minutes of reverse transcription.

These results demonstrate a genome-length-dependent decline in core elasticity during reverse transcription, with WT cores exhibiting the highest susceptibility to structural failure. This finding indicates that additional factors, other than genome length, likely play a role in the pronounced mechanical changes observed in WT cores during reverse transcription.

## Discussion

Previous studies demonstrated that reverse transcription serves as a mechanical driver of HIV-1 uncoating, wherein the conversion of flexible single-stranded RNA (ssRNA) into rigid double-stranded DNA (dsDNA) generates internal pressure that leads to structural disassembly of the viral core [19,20]. This mechanistic model highlights the importance of reverse transcription progression in destabilizing the capsid lattice. However, the minimum amount of synthesized viral genome required to initiate or complete this process was unclear.

The current findings build upon this model by investigating the impact of genome length on uncoating. The results reveal that the synthesis of a 4 kb DNA, although sufficient to partially destabilize the core, is inadequate to induce its complete disassembly. In contrast, transcription of 6 kb or longer genomes consistently led to nearly complete disassembly of the core, with WT cores (9.2 kb genome) showing the highest degree of disassembly. These observations are consistent with another recent study demonstrating that a genome exceeding 3.5 kb in length is necessary to induce significant capsid destabilization during infection [12].

The relationship between reverse transcription progression and core brittleness observed here suggests a critical genome-length threshold for productive uncoating. The gradual increase in dsDNA synthesis likely enhances capsid pressure and disrupts lattice integrity through cumulative structural strain. Our data suggest that weakening of the core structure initiates prior to the completion of reverse transcription. Consequently, the susceptibility of the HIV-1 core to structural failure in response to externally applied force progressively increases as reverse transcription advances. Notably, cores containing shorter genomes, such as the 3.7 kb variant, exhibited partial damage following compression but retained some structural integrity, indicating insufficient mechanical force to fully destabilize the lattice. This threshold-dependent behavior underscores the finely tuned balance between capsid rigidity and elasticity required for regulated disassembly, further emphasizing the interplay between genome length and mechanical perturbations.

Intriguingly, the volume recovery measured at the local compression site was not significantly affected by the progression of reverse transcription. This observation underscores a fundamental distinction between the local and global mechanical properties of the HIV-1 core. The region subjected to high-force compression was located within the central body of the core, a structurally robust domain that remains largely intact even as reverse transcription proceeds. Nevertheless, the mechanical stress induced by localized compression is expected to propagate throughout the capsid lattice, potentially reaching distant regions of the core. If these distal regions have accumulated structural defects or fractures—such as those increasingly observed during reverse transcription, the core is more likely to break at those vulnerable sites rather than at the intact compression zone. The lack of a clear correlation between volume recovery and reverse transcription progression indicates that the local capsid lattice architecture, and by extension its elastic response to deformation, remains unchanged. These findings support the notion that reverse transcription does not alter the intrinsic mechanical properties of the local lattice but rather compromises structural integrity at larger scales through fracture formation.

The HIV-1 core has long been recognized as more than a simple container for the viral genome and enzymes, with its material properties playing a pivotal role in its physical behavior. We recently demonstrated that the HIV-1 core possesses exceptional elasticity, enabling it to withstand compressive forces without sustaining structural damage [36]. Importantly, this elasticity can be significantly reduced through specific mutations in the capsid protein, which alter the mechanical properties of the core and interfere with nuclear entry. These findings underscore the molecular determinants of core elasticity, offering insights into how capsid protein mutations modulate mechanical properties and structural responses to external forces.

The findings of the current study highlight the importance of maintaining core structural integrity to preserve the high elasticity of the core. During early stages of reverse transcription, the core structure remains largely intact, preserving its high elasticity. This mechanical resilience is progressively diminished as reverse transcription advances, in agreement with earlier studies showing the formation of small fractures in the capsid lattice [9,22]. These structural defects initially reduce core elasticity while still maintaining sufficient mechanical stability to withstand compression. However, at later stages of reverse transcription, the core undergoes pronounced disassembly, leading to a substantial loss of elasticity. At this stage, cores that are subjected to compressive forces largely collapse, reflecting a critical mechanical transition linked to advanced reverse transcription.

A similar trend was observed in the context of genome length. Cores containing smaller genomes retained high elasticity and structural integrity following reverse transcription, whereas longer genomes induced more extensive damage to the capsid lattice, resulting in a marked reduction in elasticity. This genome -length -dependent decrease in core elasticity emphasizes the interplay between internal capsid pressure, structural damage, and mechanical resilience.

The extreme elasticity of the HIV-1 core has also been proposed to play a crucial role in nuclear entry. Specifically, core elasticity facilitates prolonged docking at the nuclear pore complex (NPC), enabling successful nuclear entry and subsequent infectivity [36]. In the current study, core elasticity remained high during the early stages of reverse transcription, a period when the core is likely traversing the cytoplasm and approaching the nuclear envelope. However, as reverse transcription progressed, the elasticity of the core declined significantly, reaching levels that could hinder nuclear entry.

These results suggest the existence of a biological timer that begins with the initiation of reverse transcription, likely upon core entry into the target cell cytoplasm. For successful infection, the core must reach the nuclear envelope and dock at the NPC before reverse transcription progresses to a stage where core elasticity is insufficient for nuclear entry. This requirement establishes a time window of approximately two hours post-entry, during which the core must traverse the cytoplasm and arrive at the nuclear envelope while retaining sufficient elasticity. Notably, previous studies have demonstrated that the HIV-1 core reaches the nucleus within 1–2 hours following cellular entry [40,41], aligning with this proposed time constraint. Furthermore, the correlation between the length of the reverse-transcribed dsDNA, the duration of transcription, and core elasticity supports the hypothesis that nuclear import precedes the completion of reverse transcription [8,14,27,29,30,42]. Together, these findings reinforce the model wherein the mechanical properties of the HIV-1 core are temporally regulated to facilitate successful nuclear entry and subsequent integration into the host genome.

## Materials and methods

### HIV-1 virus production and core purification

HIV-1 pseudoviruses were produced in HEK 293T cells following established protocols [20]. Briefly, cells were transfected with 2 μg of Env-defective IN- variants of the full-length HIV-1 construct R9 (WT or CA-/RT-mutant viruses) using 10 μg of polyethyleneimine (PEI; Sigma-Aldrich). Virus-like particles (VLPs) were generated by transfecting cells with 2 μg of psPAX2 plasmid. For experiments requiring different genome lengths, plasmids encoding genomes of 3.7 kb, 6.0 kb, and 10.0 kb were co-transfected with psPAX2. After 18–20 hours, the growth medium was replaced, and the virus-containing supernatant was collected, filtered, and concentrated via ultracentrifugation over a 60% OptiPrep cushion. The virus pellet was resuspended in TNE buffer and further concentrated using centrifugal concentrators.

Cores were isolated from the virus-containing supernatant using a previously described protocol [20]. HIV-1 particles were treated with 1% Triton X-100 in MOPS buffer (pH 7.0) supplemented with IP6 and centrifuged. The resulting pellet was washed and resuspended in MOPS buffer. Freshly isolated cores were used for atomic force microscopy (AFM) measurements. Reverse transcription was initiated by adding MgCl2 and dNTPs.

### AFM sample preparation

AFM samples were prepared following established protocols [20]. Isolated HIV-1 cores were incubated on hexamethyldisilazane (HMDS)-coated glass slides in a humid chamber at room temperature. AFM measurements were conducted on the adhered sample without fixation. Each experiment was repeated a minimum of three times, with independently purified pseudoviruses. All measurements were carried out using a JPK Nanowizard Ultra-Speed atomic force microscope (JPK Instruments, Berlin, Germany) mounted on an inverted optical microscope (Axio Observer; Carl Zeiss, Heidelberg, Germany). Silicon nitride probes with a mean cantilever spring constant of 0.12 N/m (DNP, Bruker, Germany) were employed. Topographic images were captured in quantitative imaging (QI) mode at a speed of 0.5 lines/s and a loading force of 300 pN and then processed using WSxM software (Nanotec Electronica).

### Core elasticity measurements and analysis

Core elasticity was evaluated by measuring volume recovery following compression [36]. Atomic force microscopy (AFM) was operated in Quantitative Imaging (QI) mode using a low indentation force of 300 pN. A small rectangular region on the core’s main body was selected for analysis and scanned. Compression was applied by rescanning this region with a maximum loading force of 5 nN. Following compression, the force was reduced to 300 pN, and the region was repeatedly imaged for 5–6 minutes. The entire core was then reimaged at 300 pN to evaluate structural changes. Image processing was conducted using a custom MATLAB script to calculate the volume within the selected region. The initial pre-compression volume was normalized to 100%, with subsequent volumes expressed as percentages relative to this value. Volume recovery was plotted over time. Structural integrity was assessed by comparing pre- and post-experiment core images, and elasticity was estimated by determining the percentage of intact versus broken cores after forced compression.

## Supporting information

S1 Data Raw data. (XLSX)
